# Targeted Drugs as Maintenance Therapy after Autologous Stem Cell Transplantation in Patients with Mantle Cell Lymphoma

**DOI:** 10.3390/ph10010028

**Published:** 2017-03-10

**Authors:** Fengting Yan, Ajay K. Gopal, Solomon A. Graf

**Affiliations:** 1 Department of Medicine, University of Washington, Seattle, WA 98195, USA; yfengtin@fredhutch.org (F.Y.); agopal@uw.edu (A.K.G.); 2 Fred Hutchinson Cancer Research Center, Seattle, WA 98109, USA; 3 Veterans Affairs Puget Sound Health Care System, Seattle, WA 98108, USA

**Keywords:** stem cell transplantation, mantle cell lymphoma, maintenance therapy

## Abstract

The treatment landscape for mantle cell lymphoma (MCL) is rapidly evolving toward the incorporation of novel and biologically targeted pharmaceuticals with improved disease activity and gentler toxicity profiles compared with conventional chemotherapeutics. Upfront intensive treatment of MCL includes autologous stem cell transplantation (SCT) consolidation aimed at deepening and lengthening disease remission, but subsequent relapse occurs. Maintenance therapy after autologous SCT in patients with MCL in remission features lower-intensity treatments given over extended periods to improve disease outcomes. Targeted drugs are a natural fit for this space, and are the focus of considerable clinical investigation. This review summarizes recent advances in the field and their potential impact on treatment practices for MCL.

## 1. Introduction

Mantle cell lymphoma (MCL) is an uncommon and heterogeneous subtype of B-cell non-Hodgkin lymphoma (B-NHL). It arises from antigen-naïve B-cells that proliferate in the mantle zone of lymph node germinal centers, and typically presents in an advanced stage, involving lymph nodes and extranodal sites including the gastrointestinal tract. MCL cells are small-to-intermediate sized B-cells that usually overexpress cyclin D1 due to t(11;14) chromosomal translocation [1]. Though B-NHLs are generally classified as either aggressive or indolent—a distinction that informs their natural history and treatment goals—MCL straddles this division. With important exceptions, MCL behaves clinically aggressively; however, like other indolent B-NHLs it is generally considered incurable and treatments are given to palliate symptoms and prolong survival.

Upfront intensive treatment for MCL aims to achieve deep—and consequently, long-term—remissions. After important advances over the past two decades, contemporary regimens include the monoclonal antibody rituximab and high-dose cytarabine combined with multi-agent chemotherapy backbones such as cyclophosphamide, doxorubicin, vincristine, and prednisone (CHOP) [2]. For younger, fit patients that achieve remission with chemoimmunotherapy, consolidation therapy is recommended. This consists of myeloablative high-dose chemo- and/or radiation-therapy (HDT) requiring rescue of the bone marrow stem cell compartment with autologous stem cell transplantation (SCT). Median time to treatment failure and disease relapse using this approach may exceed 7 years [3,4]. Disease relapse is nevertheless inevitable, and is often characterized by more treatment-resistance; therefore, improvement in standard therapies is needed.

Maintenance therapy—herein defined as relatively low intensity treatment given for extended periods to patients in remission—has received considerable interest in the post-autologous SCT setting for MCL. Maintenance therapy is well-established in other oncological settings, including patients with MCL who receive upfront rituximabCHOP (R-CHOP) without autologous SCT consolidation [5]. As a general rule, maintenance therapy should improve disease-specific outcomes while minimizing toxicities, including physical, financial, and logistical. A growing number of novel biologically-targeted therapies are profoundly altering the landscape of MCL treatment options in both first-line and relapsed settings [6,7]. These agents act through a variety of mechanisms, and possess unique and often limited toxicity profiles; as such, they make attractive candidates for post-autologous SCT maintenance therapy. This review examines the role of targeted therapies to improve outcomes after autologous SCT for MCL.

## 2. Autologous Stem Cell Transplantation for Mantle Cell Lymphoma

While its benefit in contemporary treatment algorithms is not established by randomized phase III data, HDT and autologous SCT consolidation is widely accepted as the optimal therapy for fit patients with MCL in remission. It is reserved for patients younger than 65–70 years old, though no strict chronologic age limit exists. As MCL incidence increases with age, approximately half of newly diagnosed patients may prove eligible for high-intensity treatment. The approach is supported by several studies showing favorable outcomes [8,9,10,11,12]. HDT and autologous SCT carries risk of treatment-related mortality on the order of approximately 1%–5%, depending on clinical variables, as well as long-term morbidity including an increased risk for secondary malignancies. The assumed superiority of this strategy must therefore be continually re-evaluated in this era of ever-expanding treatment options for MCL. For now, it remains the standard of care and efforts are ongoing to improve its safety and efficacy.

### 2.1. Minimal Residual Disease and Pre-Emptive Treatment

Minimal residual disease (MRD) refers to detectable MCL cells in peripheral blood or bone marrow using highly sensitive methodologies, including multi-parametric flow cytometry or real-time quantitative polymerase chain reaction [13]. In patients who achieve clinical complete remission according to standard criteria (diagnostic fluorodeoxyglucose-positron emission tomography (PET)/computed tomography (FDG-PET/CT) and morphologic evaluation of the bone marrow), assessment of MRD provides additional prognostic risk stratification [3,14,15,16]. MRD assessment has been used by some to guide pre-emptive therapy; e.g., with rituximab after standard treatment, including autologous SCT consolidation [17,18]. However, MRD measurement to date remains non-standardized, and as such, is not commonly used to guide treatment selection outside of clinical protocols. At present, for patients with MCL in remission after autologous SCT, administration of maintenance therapy is the key treatment decision faced by their clinical team.

### 2.2. Maintenance Therapy

Maintenance therapy is a well-established approach to postponing disease progression in patients with indolent B-NHL after first-line multi-agent chemoimmunotherapy [19,20,21,22,23]. In addition, maintenance rituximab (MR) given every 2 months to patients with MCL in remission after induction R-CHOP without HDT and autologous SCT consolidation results in improved progression-free and overall survival compared with interferon alpha [5]. MR after induction with other lower-intensity regimens—such as the commonly-used bendamustine plus rituximab (BR) combination—has shown no clear survival benefit to date [24]. Further maturation of these data is awaited, however, and the strategy continues to be quite routinely implemented in practice. However, maintenance therapy in B-NHL can be a double-edged sword, as attempts at improving outcomes must be weighed against toxicities. In the post-autologous SCT space, toxicities can be magnified: physiologic reserve is compromised, leading to more immediate and severe drug toxicities, and financial and social support resources are often already strained.

## 3. Targeted Maintenance Therapies after Autologous Stem Cell Transplantation for Mantle Cell Lymphoma

### 3.1. Anti-CD20 Monoclonal Antibodies

MCL is characterized by the expression of the B-cell markers, including CD20—a surface B-cell antigen that regulates cell cycle initiation. Rituximab—a mouse/human chimeric IgG1-κ monoclonal antibody—targets CD20 and depletes B-cells through the activation of complement-dependent and antibody-dependent pathways. Rituximab is labeled by the Food and Drug Administration (FDA) to treat a variety of diseases, including B-NHL, chronic lymphocytic leukemia, and certain autoimmune conditions (e.g., rheumatoid arthritis). Rituximab added to CHOP improves response rate and time to treatment failure in newly-diagnosed MCL [25], and anti-CD20 antibody therapy is a component of most MCL treatment regimens today.

Rituximab monotherapy as maintenance is conceptually attractive due to a favorable side effect profile and pharmacokinetics that permit relatively infrequent dosing. Several small non-comparative studies suggested the value of maintenance rituximab (MR) after autologous SCT in patients with MCL [26,27,28]. A single-center retrospective analysis of 72 patients by Dietrich et al. reported an improvement in progression-free survival (PFS) but not in overall survival (OS) in the 22 patients that received MR administered at 375 mg/m^2^ every 3 months for 2 years (Table 1) [29]. In a cohort of 157 patients with MCL treated with HDT and autologous SCT consolidation at the Fred Hutchinson Cancer Research Center in Seattle, 50 were given post-transplant MR, and follow-up data was available for a median of 5 years [30]. In this analysis, MR was associated with an improvement in PFS (hazard ratio, HR = 0.44; *p* = 0.007) and OS (HR = 0.46; *p* = 0.03) after multivariate adjustment. Importantly, MR was given according to a variety of dosing schedules and times after autologous SCT, resulting in a relatively heterogeneously treated population, potentially enhancing the applicability of the observed results to general practice.

The French LyMa study (NCT00921414) is a large randomized phase III study investigating MR after autologous SCT. Induction chemoimmunotherapy consists of four courses of rituximab, dexamethasone, high-dose cytarabine, platinum (R-DHAP) followed by HDT and autologous SCT for patients in remission; patients not in remission after R-DHAP could receive additional R-CHOP therapy before HDT and autologous SCT. Two hundred and forty patients completed autologous SCT and were randomized 1:1 to MR, dosed at 375 mg/m^2^ every 2 months for 3 years, or no MR. The final results of the study presented at the 2016 American Society of Hematology congress showed that patients receiving MR had improved PFS (HR 0.4, *p* = 0.0007) and OS (HR 0.5, *p* = 0.0454) [31].

In the post-autologous SCT setting, hematopoeisis is weakened and prolonged cytopenias and secondary hypogammaglobulinemia resulting in impaired immunity are common. Rituximab may contribute to each process, and severe neutropenia has been observed at up to twice the rate (34% vs. 18%, *p* = 0.04) in patients receiving MR after autologous SCT [30]. Though rituximab-related neutropenia may lead to dose-delays and increase risk for infectious complications, it is typically correctable with myeloid growth factor support and does not appear to result in non-relapse mortality rates. Similarly, recurrent infections associated with hypogammaglobulinemia may be mitigated with periodic intravenous immunoglobulin support [34]. Another concern is the impact (if any) of MR on the efficacy of post-autologous SCT re-vaccination [35]. Further study and elucidation of this potential complication is needed.

Though formal publication of the LyMa study is awaited and may reveal additional considerations about the general applicability of this strategy, MR after autologous SCT appears to confer a survival advantage to patients with MCL. It has therefore been adopted as standard of care in our practice with the caveats that patients are engaged in careful counseling of potential infectious toxicities and financial support is in place; rituximab may cost in excess of 5000 U.S. dollars (USD) per dose, and a standard course of MR according to the LyMa protocol consists of up to 18 doses.

Newer anti-CD20 monoclonal antibodies are rapidly gaining traction in the management of B-NHL. Ofatumumab and obinutuzumab are fully humanized anti-CD20 antibodies that target CD20 epitopes distinct from rituximab’s, and have proven efficacy in certain B-NHLs including chronic lymphocytic leukemia and follicular lymphoma [36,37,38,39]. Little published data exists on their use in MCL [40,41], but preclinical data is promising [42,43]. While no data are available to recommend their use in the post-autologous SCT maintenance setting, a multicenter phase II LyMa study (NCT02896582) is planned for patients with MCL using obinutuzumab-DHAP induction followed by HDT and autologous SCT consolidation with 3 years of obinutuzumab maintenance then additional obinutuzumab maintenance on demand according to MRD status. This trial anticipates enrollment of 83 patients with primary completion estimated for 2019.

### 3.2. Radioimmunotherapy

Radioimmunotherapy combines the lymphotoxic properties of radiation with specific immunologic targeting. The approach has been quite extensively studied in MCL, including as conditioning therapy prior to autologous SCT in the relapsed or refractory setting and as late intensification after first incomplete remission [10,44,45]. Ibritumomab tiuxetan—a murine monoclonal anti-CD20 antibody joined to a radioactive isotope yttrium-90 with the chelator tiuxetan—is approved for indolent B-NHL in the salvage and consolidation settings. It is expensive, with a single dose potentially costing upwards of 50,000 USD, and relatively unwieldy to use in routine practice due to required coordination with nuclear medicine or radiation oncology teams. Nevertheless, it features a unique mechanism of action and can be delivered safely and efficiently by experienced clinicians.

Mondello et al. performed a retrospective study on ibritumomab tiuxetan administered after autologous SCT in 57 patients with MCL treated in Italy and Austria [32]. All patients were treated with three cycles of R-CHOP then three cycles of R-DHAP induction followed by HDT and autologous SCT. Six to ten weeks after transplant, only the 28 patients at the Italian site received further consolidation with ibritumomab tiuxetan. Rituximab maintenance, consisting of 375 mg/m^2^ administered every 12 weeks for 2 years, was given to all patients. Intensification of consolidation with ibritumomab tiuxetan was associated with prolonged PFS (not reached versus 7 years, *p* = 0.001) and OS (not reached versus 8.1 years, *p* = 0.008), and appeared to attenuate the prognostic significance of the Mantle Cell Lymphoma International Prognostic Index (MIPI) score. Ibritumomab tiuxetan was well tolerated, with the key toxicities of grade 3–4 neutropenia in 11% and grade 3–4 thrombocytopenia in 18%. As a single consolidation-intensification dose, ibritumomab tiuxetan may ultimately prove valuable as “pre-maintenance” followed by anti-CD20 immunotherapy maintenance, particularly in highest-risk MCL, but further investigation is required.

### 3.3. Proteosome Inhibitors

Bortezomib is a small molecule that reversibly binds and inhibits the 20S proteasome, resulting in cellular apoptosis via a variety of mechanisms. In MCL, the activity of bortezomib is primarily attributed to decreased proteasome degradation of inhibitor of kB (lkB) allowing continued inhibition over NF-κB pathway activity—a critical element in MCL tumorigenesis [46]. Bortezomib has proven efficacy in MCL both as a single agent and in combination with chemoimmunotherapy backbones: it received FDA approval for relapsed MCL in 2006 and in first-line therapy in 2014 [47,48,49]. Bortezomib also has promising activity as single-agent maintenance given after induction R-CHOP for MCL, with 65 patients showing an impressive 2-year PFS of 62% in the single-arm phase II SWOG S0601 study [22].

As maintenance therapy after autologous SCT, bortezomib was evaluated in the CALGB (Alliance) 50403 study [33]. Patients were administered induction therapy with two to three cycles of augmented R-CHOP (increased dose of cyclophosphamide and addition of methotrexate) followed by high-dose stem-cell chemomobilization with cytarabine, etoposide, rituximab, and filgrastim and then HDT and autologous SCT. After two doses of post-transplant rituximab, patients were randomized to bortezomib consolidation (1.3 mg/m^2^ intravenous infusion on days 1, 4, 8, and 11 of a 3-week cycle for four cycles total) or bortezomib maintenance (1.6 mg/m^2^ intravenous infusion weekly 4 of 8 weeks for 18 months) beginning on approximately day 90 after transplant. One-hundred and fifty-one patients were enrolled, and 118 underwent autologous SCT. Of these, 102 were randomized to bortezomib consolidation or bortezomib maintenance. The 2-year PFS rates in the bortezomib maintenance and bortezomib consolidation arms were similar (84% and 89%, respectively), but bortezomib consolidation was associated with relatively more toxicity. Compared with CALGB 59909 (which consisted of the identical regimen without bortezomib maintenance or consolidation and thus served as an historical control), CALGB 50403 demonstrated improved 5-year PFS rates from time of transplantation (72.7% versus 51.5%, *p* = 0.0006), suggesting a PFS benefit for post-autologous SCT bortezomib maintenance in MCL [50].

The critical toxicity with prolonged bortezomib use is peripheral neuropathy, which can become severe and long-lasting if not recognized. Prior treatment with neurotoxic chemotherapeutic agents appears to increase the incidence of neuropathy from bortezomib [51]. In CALGB 50403, adverse events—namely neuropathy and cytopenias—associated with bortezomib led to withdrawal from the study in 13% of patients treated on the maintenance regimen. The maintenance schedule was also notably relatively burdensome from the perspective of time commitment, with a total of approximately 36 infusions administered over the course of the 18 months. Financial toxicity is another important consideration, as a course of four doses of bortezomib may cost between 5000 and 10,000 USD.

Later generations of proteasome inhibitors include carfilzomib, an irreversible inhibitor of the 20S proteasome subunit, and ixazomib, an orally administered and reversible 20S proteasome subunit. Each have somewhat less associated neurotoxicity than bortezomib and are established therapies in treating multiple myeloma. Carfilzomib has preclinical data supporting its use in MCL [52], and a phase I study (NCT01926665) is planned evaluating the maximum tolerated dose of 6 months carfilzomib after autologous SCT for MCL and other lymphomas. Ixazomib is currently being tested in a phase I/II study (NCT02632396) post-autologous SCT maintenance, with a single oral dose given on days 1, 8, and 15 of 28 day cycles for up to ten cycles. The results of these trials may provide rationale for further study that ultimately expands post-autologous SCT maintenance options for MCL.

### 3.4. Bruton’s Tyrosine Kinase Inhibitor

Bruton’s tyrosine kinase (BTK) is important to B-cell signaling, maturation, and to mantle cell lymphomagenesis through activation of the Ras/RAF/MEk/ERK and NF-κB pathways [53]. Ibrutinib covalently binds BTK, irreversibly inactivating the kinase. The activity of ibrutinib in MCL was established in a phase II trial in 111 patients with relapsed or refractory disease, of whom 67% responded to ibrutinib monotherapy (21% complete response rate) [54]. Ibrutinib is FDA approved for use in patients that have received at least one prior therapy. Ibrutinib is a once-daily oral drug with a favorable side effect profile, making it an attractive candidate for maintenance therapy.

NCT02242097 is a single-arm phase II trial of up to 4 years of continuous ibrutinib maintenance therapy after first-line treatment of MCL. Interestingly, eligible induction treatment regimens include both multi-agent chemoimmunotherapies with or without autologous SCT. The TRIANGLE study is a randomized, three-arm, parallel-group, open label, international phase III study with planned accrual of 870 patients being conducted by the European MCL Network. The study involves upfront therapy with six alternating courses of R-CHOP and R-DHAP followed by autologous SCT (control arm A) versus R-CHOP + ibrutinib alternating with R-DHAP followed by autologous SCT and up to 2 years of ibrutinib maintenance (experimental arm A + I) versus R-CHOP + ibrutinib alternating with R-DHAP followed by up to 2 years of ibrutinib maintenance (experimental arm I), with a primary endpoint of failure-free survival. The TRIANGLE study is actively recruiting patients, and its estimated primary completion is for 2021.

Ibrutinib is associated with toxicities including cytopenias, gastrointestinal symptoms, edema, and musculoskeletal symptoms, but these are typically mild [55]. Risk of bleeding seems to be increased with ibrutinib due to off-target effects on platelet function, and concurrent use of vitamin K antagonist anticoagulation is generally advised against [56]. This can complicate management—particularly as ibrutinib is also associated with an increased risk of atrial fibrillation. Nevertheless, ibrutinib is generally extremely well-tolerated, even in frail individuals. Its potential use in MCL as long-term treatment in the post autologous SCT maintenance setting will certainly have to take its cost into account, which is on the order of 10,000 USD a month.

### 3.5. Lenalidomide

Lenalidomide—an analogue of thalidomide—was FDA approved in 2013 for the treatment of relapsed/refractory MCL based on phase II data showing an overall response rate of 28% (CR of 8%) and median duration of response of 16.6 months in 134 patients with relapsed or refractory disease [57]. Its mechanism of action on lymphoma involves engagement of the unbiquitin ligase cereblon resulting in both immunomodulation of the tumor microenvironment—particularly natural killer cell stimulation—and direct tumor killing [58].

Fondazione Italiana Linfomi is an ongoing randomized phase III study opened in 2010 and that has now completed recruitment (NCT02354313). It is evaluating the role of lenalidomide maintenance after upfront autologous SCT in MCL. The study is designed to randomize 300 subjects to lenalidomide maintenance dosed at 10–15 mg daily on days 1–21 out of every 28 for 2 years versus observation after an induction of three cycles of R-CHOP followed by high-dose cyclophosphamide and two cycles of high dose cytarabine then HDT and autologous SCT consolidation. The primary endpoint is PFS at 30 months after randomization.

Similar to ibrutinib, lenalidomide is a once-daily medication taken orally. However, it is usually associated with more significant toxicities—particularly fatigue and cytopenias requiring dose adjustments or delay. In addition, it increases the risk of arterial and venous thromboembolic events, and concurrent prophylaxis with, for example, a daily aspirin should be administered. Lenalidomide is also associated with a small increased risk of secondary malignancies in patients receiving it for multiple myeloma, though whether that applies to other situations is not known. It is also a high-cost medication. Overall, lenalidomide as maintenance therapy after autologous SCT for MCL may prove efficacious, but other options as described above are likely to eclipse it due to its relatively more significant toxicity profile.

### 3.6. Combination Treatments

Various studies are exploring the role of combination therapies for post-autologous SCT maintenance for MCL. These include NCT01267812, a phase II multicenter trial using bortezomib plus rituximab in 36 patients with MCL and NCT00992446, a phase II study of bortezomib and vorinostat, a histone deacetylase inhibitor, in 20 patients with NHL, including MCL. Both studies are active, and recruitment for NCT01267812 is ongoing. NCT01045928 was a phase I/II trial of the combination of lenalidomide with rituximab as maintenance after autologous SCT in patients with B-NHL that was terminated because of toxicity observed in phase I.

## 4. Conclusions

The introduction of targeted therapies has revolutionized the treatment paradigm of MCL. Based on recently presented prospective data from LyMa, post-autologous SCT MR should now be considered standard of care, but requires attention to its potential impact on immune reconstitution and cytopenias. Other agents are under intensive investigation as maintenance after autologous SCT, and will hopefully prove efficacious in their own right, adding options featuring non-overlapping toxicity profiles. Results of these studies are anticipated in the coming years and will likely guide further research efforts, including MRD-stratification of initiation and duration of treatment, biomarker-based selection of therapy, and rational therapeutic sequencing or combinations.

## Figures and Tables

**Table 1:** Selected studies evaluating maintenance therapy after autologous stem cell transplantation for mantle cell lymphoma.

Reference	Year Reported	Post-Autologous SCT Intervention	Study Type	Patients	Key Results of Intervention
Dietrich et al. [29]	2014	R every 3 months for 2 years	Retrospective	72	PFS (HR 0.23) but not OS improved after multivariate adjustment
Graf et al. [30]	2015	Variable schedule of R	Retrospective	157	Both PFS (HR 0.44) and OS (HR 0.46) improved after multivariate adjustment
Le Gouill et al. [31]	2016 (abstract)	R every 2 months for 3 years	Randomized phase III	240	Both PFS (HR 0.4) and OS (HR 0.5) improved
Mondello et al. [32]	2016	R every 12 weeks for 2 years +/− prior ibritumomab tiuxetan	Retrospective	57	Both PFS (median not reached versus 7 years) and OS (median not reached versus 8.1 years) improved with ibritumomab tiuxetan
Kaplan et al. [33]	2015 (abstract)	2 doses of R then bortezomib	Randomized phase II	102	5-year PFS of 72.7% improved over historical control (51.5%) not administered post autologous SCT bortezomib
Fondazione Italinana Linfomi	Ongoing	Lenalidomide for 2 years	Randomized phase III	300 (planned)	Recruitment completed
TRIANGLE	Ongoing	Ibrutinib for 2 years	Randomized phase III	870 (planned)	Estimated completion 2021

Abbreviations. SCT, stem cell transplantation; PFS, progression free survival; OS, overall survival; HR, hazard ratio; R, rituximab.
